# Comparative effectiveness of a multimodal intervention package on surface cleaning and disinfection in Brazilian neonatal intensive care units

**DOI:** 10.3389/fpubh.2025.1557538

**Published:** 2025-05-06

**Authors:** Maria Heloisa do Nascimento Silva, Caroline Lopes Ciofi-Silva, Verusca Soares de Souza, Daniel de Macêdo Rocha, Alvaro Francisco Lopes de Sousa, Adriana M. S. Felix, Aires Garcia dos Santos Júnior

**Affiliations:** ^1^Universidade Federal de Mato Grosso do Sul, Campus de Três Lagoas, Três Lagoas, Mato Grosso do Sul, Brazil; ^2^School of Nursing, Universidade Estadual de Campinas (UNICAMP), Campinas, São Paulo, Brazil; ^3^Universidade Estadual do Paraná, Paranavaí, PR, Brazil; ^4^Universidade de Mato Grosso do Sul, Campus Coxim, Coxim, Mato Grosso do Sul, Brazil; ^5^Instituto de Ensino e Pesquisa, Hospital Sírio-Libanês, São Paulo, SP, Brazil

**Keywords:** disinfection, infection control, training programs, personal satisfaction, work performance

## Abstract

**Objective:**

To compare the impact of a package of interventions on surface cleaning and disinfection in two public and private neonatal intensive care units (NICUs).

**Method:**

This is a quasi-experimental, quantitative study. The study consisted of three phases: baseline (phase I), implementation of the intervention package without feedback to the team (phase II), and, finally, short-term monitoring with feedback to the team (phase III). A total of 864 evaluations were carried out according to the monitoring methods: adenosine triphosphate, visual inspection, microbial load counting, and detection of Staphylococcus aureus and testing for its methicillin resistance (MRSA) in each unit over 4 months, evaluating six high-frequency touch surfaces, before and after cleaning and disinfection carried out by nursing and sanitizing professionals.

**Results:**

When comparing the effect of the package in the two units (public and private), no significant differences were found in the proportions of MRSA-positive surfaces in all the phases evaluated. The same occurred concerning the ATP method, which showed no significant differences between the hospitals in all study phases. Concerning the microbial count, in phase II of the study, only on one surface (scales) was a significantly lower difference found in the private hospital compared to the public one. Visual inspection indicated that the private NICU had a substantially higher proportion of surfaces with adequate hygiene in phase I: the infusion pump and the armchair; in phase II, the counter and in phase III again the counter surface. Concerning human factors, when comparing the two institutions, there were no statistically significant associations or correlations with job satisfaction. However, the public institution had higher work performance scores than the private one.

**Conclusion:**

The study highlights that the rigorous implementation of intervention packages for cleaning in NICUs, even though they are different, still showed similar results in terms of effectiveness for all the methods used, except visual inspection. This study showed that even though the employees had various levels of work performance, there was still a similar effect on the outcome of the intervention package.

## Introduction

Healthcare-associated infections (HAIs) pose a serious threat to patient safety worldwide, leading to increased morbidity, mortality, and hospital costs. The complexity of cases, particularly in intensive care units, contributes to adverse clinical outcomes (1, 2).

Infections are a critical issue in Neonatal Intensive Care Units (NICUs), affecting ~30% of newborns and contributing to up to 40% of neonatal deaths in developing countries. Therefore, surveillance and control measures are essential to reducing morbidity and mortality, including hand hygiene, rational antibiotic use, standard precautions, and control of environmental contamination (3, 4).

To prevent the transmission of HAIs, it is crucial to adopt rigorous cleaning and disinfection (C&D) practices, which involve the removal of visible dirt and the use of effective disinfectants on high-contact surfaces. These measures are essential for protecting public health and reducing infection risks (5). Despite this, shortcomings in C&D practices persist (6). To ensure the effectiveness of C&D processes, it is necessary to implement quality monitoring methods that guide protocol adjustments and staff training. Several methods are available, including visual inspection, quantitative and qualitative microbiological analysis (identification of microorganism species), and the quantification of adenosine triphosphate (ATP) (7).

Visual inspection is a simple and low-cost method; however, it does not provide an objective assessment of cleanliness. In contrast, microbial load testing quantifies the presence of microorganisms on surfaces by measuring colony-forming units (CFU), but this method is more expensive, time-consuming, and requires specialized laboratory facilities (7, 8). One epidemiologically relevant species is MRSA.

Another commonly used method is ATP bioluminescence testing, which is a simple, rapid, and quantitative approach. Initially applied in the food industry, ATP assays have recently been adopted in healthcare settings to monitor the cleanliness of environmental surfaces (9, 10).

The C&D process is primarily carried out by nursing professionals (NPs) and hygiene professionals (HPs). To ensure compliance with cleaning standards, the active engagement of these professionals is essential. Understanding organizational culture-related factors, such as the commitment and work performance of NPs and HPs, not only enhances employee wellbeing but also improves the quality of care provided (11, 12).

Educational strategies, feedback mechanisms, and the standardization of practices and supplies are widely employed to strengthen adherence to C&D protocols. A systematic review found that studies on the subject provided limited information regarding HP-related aspects, workload, the impact of training, and the frequency of object and surface cleaning per shift. The review recommended further studies with detailed evaluations of the effectiveness of interventions (13).

Despite the growing recognition of the role of environmental service workers in infection prevention, significant gaps remain in understanding the daily work dynamics of these professionals. NPs and HPs face constant challenges in an environment that demands repetitive tasks under high pressure, often in conditions that negatively impact their health and wellbeing. Although they play a crucial role in hygiene and infection control, these workers are frequently overlooked within organizational contexts and do not receive the necessary recognition. Their inclusion in interdisciplinary discussions and training initiatives is rare, limiting opportunities for skill development and appreciation of their essential contributions to public health (14).

This study contributes to the scientific literature on this topic. The research is guided by the following questions: Are there differences in the effectiveness of an intervention package when comparing two NICUs, one public and one private? What are the levels of job satisfaction and performance among the professionals responsible for the C&D process in the two NICUs?

## Objective

To compare the effect of an intervention package on surface C&D, as well as the levels of job satisfaction and performance of nursing and hygiene professionals responsible for environmental hygiene in public and private neonatal units.

## Materials and methods

### Ethical aspects

This study was approved by the institutional Research Ethics Committee under opinion number 5.961.710.

### Type of study

This is a quasi-experimental, quantitative study.

### Study site

The research was conducted simultaneously in two NICUs, one public and one private, each with 10 beds. Both NICUs had a qualified multidisciplinary team. Data collection took place from August to November 2023.

In Brazil, according to the guidelines of the Health Surveillance Agency, the responsibility for surface C&D can be shared between NPs and HPs. In this study, surfaces under the responsibility of both teams were evaluated. It is up to each institution to determine, through internal policies and procedures, the specific responsibilities of each professional (15).

### Selection of participants (inclusion and exclusion criteria)

The study included all NPs (directly hired by the institution) responsible for the C&D process in the two participating NICUs.

In the private NICU, the team consisted of 17 professionals, including 15 nursing staff and two hygiene staff, all of whom agreed to participate in the research. In the public NICU, all 29 professionals responsible for C&D participated in the study, including 25 from the nursing team and four from hygiene services. Trainees were excluded from the sample.

### Protocol and products used before the educational intervention

The institution had an established ICU sanitization protocol. However, this protocol did not specify which product should be used for each type of surface or the appropriate cleaning materials. According to the standard protocol, concurrent cleaning was recommended once a day. HPs were responsible for cleaning the ceiling, floor, shelves, and walls, while NPs were in charge of disinfecting incubators during the weekly terminal cleaning.

A preliminary observation of the C&D process was conducted over a week using a checklist adapted from CDC guidelines (2010). It was noted that each professional used a different cleaning product for surface C&D.

In the NICUs, alcohol gel dispensers were available near all beds, as well as in other locations throughout the unit. Visitors had unrestricted access to the unit, and guidance on proper hand hygiene techniques was provided at the entrance.

### Procedures

Data collection occurred between August and November 2023 and was divided into three phases: I. Baseline assessment; II. Implementation of the Intervention Package without providing feedback to the team (PI); and III. Medium-term monitoring with feedback to the team.

In phase I (baseline), detailed monitoring of the C&D process was carried out using the following methods: a) visual inspection; b) ATP measurement; c) quantitative assessment of microbial load using RODAC-type contact plates; d) detection of MRSA.

The 10 × 10 cm area proposed by the area delimiter provided by the manufacturer LaborClin (“Produtos para Laboratórios Ltd.”; Pinhais, PR, Brazil) was standardized for ATP data collection. The swab was applied to the surfaces using horizontal and vertical movements, then agitated for 10 s to ensure proper contact with the reagent before being inserted into the luminometer. A reading of < 250 relative light units (RLUs) was considered acceptable (16).

For microbial load assessment, Rodac-type contact plates (manufacturer) were used. These plates, made of tryptic soy agar, have a 24 cm^2^ surface area and hold up to 20 ml of medium. To quantify microbial load, the plates were pressed onto the surfaces for 10 s and then incubated at 37°C. CFU counts were assessed after 48 h using a stereomicroscope under reflected light (17).

For the identification of *Staphylococcus aureus*, Petrifilm™ Staph Express plates (manufacturer) were used. These plates contain a chromogenic and selective culture medium, which enables the differentiation of *S. aureus* by producing red-violet colonies (manufacturer's guide).

To detect MRSA, colonies were inoculated onto a selective culture medium (manufacturer). This medium contains inhibitors to prevent the growth of non-target bacteria and differential indicators that allow MRSA to be identified. The addition of chromogenic substrates facilitates visualization through distinct colony staining (manufacturer's guide).

In phase II (Implementation of the Intervention Package), interventions were designed based on the findings from phase I. These interventions included educational activities, standardization of practices, and selection of appropriate cleaning supplies.

After consenting to participate in the study, formalized through the Informed Consent Form (ICF), NPs and HPs completed the Work Performance and Job Satisfaction Scale, detailed below.

An educational intervention was conducted in each NICU, comprising 12 training sessions per unit, with each session lasting 1 h, over a 1-week period. Institutional protocols were revised based on the recommendations of the Centers for Disease Control and Prevention (CDC) and the National Health Surveillance Agency (ANVISA), as well as validated educational materials from worker health promotion programs (15, 18, 19).

The intervention aimed to improve the sanitization of high-touch surfaces in patient areas and locations associated with direct clinical care. A key component was training staff on thorough cleaning techniques for surfaces and equipment (20).

In collaboration with the Hospital Infection Control Committees, NICU Nursing Coordinators, and Hygiene Service Coordinators, protocols were updated to standardize concurrent cleaning twice daily and as needed (15).

The use of a specific disinfectant for floors, walls, furniture, and equipment was established. This disinfectant contains 5th-generation di-decyl quaternary ammonium compounds combined with polymeric biguanide from the chlorhexidine family (*Polyhexamethylene Biguanide*), providing broad-spectrum microbicidal activity against bacteria, yeasts, fungi, viruses, and spores. The product must be applied in a manner that ensures the surface remains moist for 10 min (21).

Additionally, the use of microfiber cloths for cleaning was standardized, as they have been shown to enhance microbial removal efficiency (22).

During the intervention, the cleaning process was adjusted, demonstrating the correct technique: when cleaning incubators and cradles, the process should begin from the inside out, always in one direction, to prevent the dispersion of dirt. If the cloth accumulates visible dirt or is used in highly contaminated areas, it should be replaced to avoid redistributing contaminants. The procedure consists of three stages, if necessary: in the first, a cloth dampened with the disinfectant solution is used to remove surface dirt; in the second, a dry cloth is applied to ensure the complete removal of any dirt residue or disinfectant remnants; and, if needed, a third application of the cloth is performed until the surface is entirely clean and free of visible residue. Photos demonstrating the correct procedure were provided to facilitate proper execution.

After this first week of team training, surface monitoring commenced immediately and continued for 30 days using the same monitoring methods applied in phase I of the study (23–25).

In phase III, surface monitoring was conducted again to evaluate the C&D process using the same methods as in phase I; however, this time, feedback was provided to the professionals. The lead researcher offered feedback on C&D practices to NPs and HPs, who were given immediate access to the assessment results, including ATP readings and visual inspections (25).

This phase lasted 30 days (23–25). The objective was to repeat the procedures to assess adherence to the implemented measures 60 days after the educational intervention.

It is important to note that in phase I, before the educational intervention, there was no **Hawthorne effect** on participants' professional practices, minimizing the possibility of external factors influencing the results. The participants were not informed about the study's objective, and sample collections followed the same protocols used for institutional testing of cleaning product effectiveness. Samples were always collected before and after the team performed the C&D process. In the subsequent phases, data collection was conducted by research team members, ensuring that the processes involved remained consistent across the different phases.

Using an intentional and non-probabilistic approach, the following items were selected for surface monitoring: the counter (marble in Unit A and wood in Unit B, used for medication preparation in both units), incubator (metal and acrylic structure in both units), armchair (polyvinyl chloride and polyester mesh), infusion pump (polycarbonate in both units), preparation table (stainless steel in both units), desk, and scale (polypropylene in both units). These items were chosen based on systematic observation, prioritizing high-touch surfaces, as recommended by the CDC (26).

Samples were collected twice weekly in each NICU, totaling eight sampling days per phase. Considering the analyzed surfaces, six samples were obtained before and six after the cleaning process, totaling 12 samples per day and 24 samples per week from each institution. Consequently, 96 samples were collected per method at the end of each month, leading to 864 evaluations conducted in each NICU, as detailed in Table 1.

**Table 1:** Evaluations conducted in each phase of the study in each NICU. Três Lagoas, MS, Brazil, 2023.

**Method**	**Stage I (4 weeks of collection)**	**Stage II (4 weeks of collection)**	**Stage III (4 weeks of collection)**	**Total evaluations**
Visual	96	96	96	288
ATP	96	96	96	288
CFU	96	96	96	288
Staphylococcus aureus	96	96	96	288
TOTAL	288	288	288	864

Source: Self-authored, 2024.

### Scales used to collect human factors data

The Job Satisfaction Scale (27) and the Performance scale developed by Vandenabeele (28) were used to collect data on satisfaction levels. The scales used to assess job satisfaction and performance levels in hospital contexts are essential for understanding professionals' perceptions. The Job Performance Scale by Vandenabeele (28), comprised of four variables, uses a Likert-type approach, offering five response options, with minimum and maximum values ranging from 4 to 20. The Job Satisfaction Scale, developed by Dépré and Hondeghem in 1995, consists of a single dimension with six variables, the answers to which are captured by a five-point Likert scale, ranging from “strongly disagree” (1) to “strongly agree” (5). Thus, the results of this scale can range from a minimum of 6 to a maximum of 30.

### Statistical analysis

The following statistical tests were applied: the Wilcoxon rank-sum test to compare the results of ATP measurements and microbial load/CFU. The Mann-Whitney test was used to compare microbial load/UFC and ATP measurements. This test uses the data median, employing a comparative and non-parametric analysis.

In addition, a quantitative analysis of the data was carried out, comparing the microbial load/CFU and ATP measurements at the different stages of the study. To achieve this, the variation in the quantitative data, which includes the total microbial load (CFU/cm^2^) and ATP, was calculated using the following expression:


(1) $$\begin{array}{c} variation\;\%\;\left(ATP\;ou\;CFU\right)=\;\frac{after-before}{before}*100. \end{array}$$

Fisher's exact test was applied when analyzing the surfaces by visual inspection in all phases to compare two proportions and check for any differences. A descriptive analysis was carried out for the variables related to job satisfaction and performance. The study adopted a significance level of 5% (*p* < 0.05).

## Results

Table 2 shows the percentages of surfaces that passed the visual inspection for both hospitals in all the phases evaluated.

**Table 2:** Results of the visual inspection of approved surfaces for both hospitals in the phases evaluated in the study.

**Visual inspection**		**Hospital**		**P-value^a^**
		**Private**	**Public**	
Phase I	Incubator	5 (62.5%)	4 (50.0%)	0.611
	Preparation table	3 (37.5%)	2 (25.0%)	0.586
	Infusion pump	6 (75.0%)	2 (25.0%)	**0.021**
	Scale	4 (50.0%)	3 (37.5%)	0.611
	Armchair	3 (37.5%)	2 (25.0%)	0.586
	Desk	6 (75.0%)	4 (50.0%)	0.285
Phase II	Incubator	3 (37.5%)	1 (12.5%)	0.228
	Preparation table	1 (12.5%)	1 (12.5%)	1.000
	Infusion pump	1 (12.5%)	0 (0.0%)	0.285
	Weight scale	3 (37.5%)	3 (37.5%)	1.000
	Armchair	3 (37.5%)	0 (0.0%)	**0.028**
	Desk	4 (50.0%)	0 (0.0%)	**0.005**
Phase III	Incubator	2 (25.0%)	0 (0.0%)	0.102
	Preparation table	1 (12.5%)	0 (0.0%)	0.285
	Infusion pump	3 (37.5%)	1 (12.5%)	0.228
	Weight scale	1 (12.5%)	4 (50.0%)	0.077
	Armchair	2 (25.0%)	0 (0.0%)	0.102
	Desk	5 (62.5%)	1 (12.5%)	**0.016**

^a^P-value referring to the test of two proportions at P < 0.05. Bold values represent those with statistical significance.

The results in Table 2 show four cases of proportions of approved surfaces that differed significantly when the hospitals were compared (*P* < 0.05). In phase I, the approval proportion for the infusion pump was substantially higher for the private than for the public hospital. The same situation occurred in phase II for the armchair and counter, showing that the approval proportion was significantly higher for the private hospital. In phase III, the desk also showed significant differences, with a higher proportion of approval for the private sector. In all critical cases, the private sector had a higher proportion of approved surfaces than the public sector, which means that the intervention was more effective in the private sector than in the public sector.

The data in Table 3 shows the results of the parameters assessed in the study comparing the hospitals.

**Table 3:** In relative light units, Adenosine Triphosphate (ATP) results for both hospitals in the phases evaluated in the study.

**ATP**		**Hospital**		**P-value^a^**
		**Median (minimum; maximum)**		
		**Private**	**Public**	
Phase I	Incubator	64.5 (11; 106)	44.5 (21; 587)	0.792
	Preparation table	104 (22; 174)	134 (15; 1,280)	0.431
	Infusion pump	78.5 (37; 342)	204 (31; 981)	0.156
	Weight scale	37 (11; 390)	68 (19; 150)	0.563
	Armchair	228 (39; 847)	390 (100; 946)	0.318
	Desk	64 (40; 1,480)	395 (31; 1,280)	0.563
Phase II	Incubator	37.5 (16; 189)	146.5 (6; 554)	0.127
	Preparation table	41 (17; 4,378)	73.5 (15; 319)	0.189
	Infusion pump	64 (24; 356)	175.5 (28; 654)	0.083
	Weight scale	77 (21; 2,703)	185 (54; 1,798)	0.127
	Armchair	341 (45; 1,843)	340.5 (60; 584)	1.000
	Desk	153 (64; 1,277)	96.5 (10; 284)	0.318
Phase III	Incubator	17 (12,38)	22.5 (10; 498)	0.563
	Preparation table	26.5 (10; 107)	26.5 (16; 61)	0.916
	Infusion pump	44 (12; 363)	33 (11; 191)	0.958
	Weight scale	26.5 (11; 361)	58 (23; 382)	0.227
	Armchair	72 (19; 447)	329 (42; 649)	0.066
	Desk	137 (20; 291)	56.5 (18; 190)	0.227

^a^P-value referring to the Mann-Whitney test at P < 0.05.

Table 3 shows no significant differences in ATP values for all study phases when comparing the hospitals evaluated. All *P*-values were higher than 0.05. Therefore, regardless of the phase evaluated, the ATP values on all surfaces in both hospitals did not differ significantly.

Table 4 shows the results of the microbial loads (CFU) compared to the hospitals.

**Table 4:** Results of microbial load/CFU for both hospitals in the phases evaluated in the study.

**Microbial load/CFU**		**Hospital**		**P-value^a^**
		**Median (minimum; maximum)**		
		**Private**	**Public**	
Phase I	Incubator	33.5 (1; 100)	24.5 (5; 56)	0.674
	Preparation table	14.5 (3; 100)	64.5 (25; 100)	0.227
	Infusion pump	32.5 (1; 100)	32.5 (3; 100)	0.752
	Weight scale	24.5 (0; 100)	25.5 (4; 65)	0.792
	Armchair	83 (48; 100)	85 (75; 100)	0.636
	Desk	15.5 (0; 100)	48.5 (3; 100)	0.344
Phase II	Incubator	14 (0; 94)	26 (0; 100)	0.494
	Preparation table	10.5 (1; 69)	27 (2; 100)	0.141
	Infusion pump	21.5 (5; 100)	83 (13; 100)	0.141
	Weight scale	19 (0; 86)	52.5 (25; 97)	**0.023**
	Armchair	65.5 (4; 100)	85 (33; 100)	0.431
	Desk	48 (6; 91)	20.5 (0; 100)	0.636
Phase III	Incubator	3.5 (1; 45)	10.5 (2; 78)	0.207
	Preparation table	22 (1; 68)	16.5 (2; 49)	0.713
	Infusion pump	27 (0; 46)	15.5 (5; 100)	0.958
	Weight scale	20 (0; 58)	17.5 (0; 126)	0.792
	Armchair	22 (0; 95)	39.5 (8; 68)	0.431
	Desk	7.5 (1, 23)	20 (0; 41)	0.127

^a^P-value referring to the Mann-Whitney test at P < 0.05. Calculation based on median. Bold values represent those with statistical significance.

According to the data in Table 4, there was one significant difference in the microbial count between the hospitals evaluated, the surface being a scale in phase II (*P* = 0.023). In this case, the microbial count was significantly lower in the private hospital than in the public one. There were no significant differences in the microbial count for the other surfaces.

Table 5 shows the results of comparing hospitals with *Staphylococcus aureus* (SA). There were no significant differences, regardless of phase.

**Table 5:** It shows the results of *Staphylococcus aureus* (SA) for both hospitals in the phases evaluated in the study.

**SA**		**Hospital**		**P-value^a^**
		**Median (minimum; maximum)**		
		**Private**	**Public**	
Phase I	Incubator	0 (0; 3)	0 (0; 33)	0.792
	Preparation table	2 (0; 13)	2 (0; 11)	0.833
	Infusion pump	0 (0; 9)	2 (0; 23)	0.674
	Weight scale	0 (0; 16)	0 (0; 0)	-
	Armchair	2.5 (0; 21)	10 (0; 26)	0.563
	Desk	0 (0; 8)	0 (0; 55)	0.833
Phase II	Incubator	0 (0; 2)	0.5 (0; 39)	0.293
	Preparation table	0 (0; 2)	0.5 (0; 27)	0.189
	Infusion pump	0 (0; 12)	1 (0; 37)	0.431
	Weight scale	0.5 (0; 7)	1 (0; 12)	0.563
	Armchair	1 (0; 12)	4 (0; 30)	0.344
	Desk	1 (0; 4)	0 (0; 38)	1.000
Phase III	Incubator	0 (0; 0)	0 (0; 6)	-
	Preparation table	0 (0; 0)	0 (0; 28)	-
	Infusion pump	0 (0; 5)	0 (0; 0)	-
	Weight scale	0 (0; 11)	0 (0; 0)	-
	Armchair	0 (0; 9)	0 (0; 9)	0.752
	Desk	0 (0; 0)	0 (0; 0)	-

^a^P-value referring to the Mann-Whitney test at P < 0.05. In some cases, the P-value was not determined due to lack of variation in the data. Calculation based on median.

Table 6 shows the percentages of MRSA-positive surfaces for both hospitals in all the phases evaluated.

**Table 6:** The results of the surfaces were positive for methicillin-resistant *Staphylococcus aureus* (MRSA) for both hospitals in the phases evaluated in the study.

**MRSA**		**Hospital**		**Valor P^a^**
		**Private**	**Public**	
Phase I	Incubator	1 (12.5%)	3 (37.5%)	0.228
	Preparation table	2 (25.0%)	4 (50.0%)	0.285
	Infusion pump	2 (25.0%)	5 (62.5%)	0.102
	Weight scale	1 (12.5%)	0 (0.0%)	0.285
	Armchair	2 (25.0%)	5 (62.5%)	0.102
	Desk	1 (12.5%)	3 (37.5%)	0.228
Phase II	Incubator	1 (12.5%)	3 (37.5%)	0.228
	Preparation table	1 (12.5%)	3 (37.5%)	0.228
	Infusion pump	2 (25.0%)	4 (50.0%)	0.285
	Weight scale	2 (25.0%)	5 (62.5%)	0.102
	Armchair	4 (50.0%)	6 (75.0%)	0.285
	Desk	3 (37.5%)	2 (25.0%)	0.586
Phase III	Incubator	0 (0.0%)	1 (12.5%)	0.285
	Preparation table	0 (0.0%)	2 (25.0%)	0.102
	Infusion pump	1 (12.5%)	0 (0.0%)	0.285
	Weight scale	2 (25.0%)	0 (0.0%)	0.102
	Armchair	0 (0.0%)	2 (25.0%)	0.102
	Desk	0 (0.0%)	1 (12.5%)	0.285

^a^P-value referring to the test of two proportions at P < 0.05.

The results in Table 6 show no significant differences in the proportions of MRSA-positive surfaces in all the phases evaluated when the hospitals were compared (*P* > 0.05). In this context, the evaluated hospitals had no significant differences regarding MRSA-positive status.

Regarding human factors, comparing the two ICUs, it was found that there was no statistically significant difference between the two units in terms of age (*p* = 0.825, Mann-Whitney test), and there was also no statistically significant difference in terms of schooling (*p* = 0.286, Mann-Whitney test). There was also no difference between the two groups regarding the length of time they had worked in the ICU (*p* = 0.430, Fisher's exact test). Concerning job satisfaction, there were no statistically significant associations or correlations when assessing the scores obtained from the 0.067 d scale (*r* = 0.19). However, the public institution scored higher on the work performance scale than the private institution (Mann Whitney W = 366.5, *p* = 0.006), as seen in Table 7.

**Table 7:** Comparison of the level of job satisfaction and performance of the professionals responsible for cleaning (nursing and hygiene) in the two institutions.

**Characteristic**	**Min-Max**	**Q1-Q3**	**Median**	**Average**	**SD**	***p*-value**
**Job satisfaction**						
Public	2.33–4.5	3.5–4	3.83	3.72	0.43	0.067 d (*r* = 0.19)
Private	2.83–4.33	3.17–3.83	3.5	3.5	0.44	
**Performance at work**						
Public	3–5	3.75–4	3.75	3.84	0.51	0.006 d (*r* = 0.29)
Private	3–4	3.25–3.5	3.5	3.46	0.31	

Comparison: p-values marked by the letter 'c' indicate performance of the Cohen's t-test and effect size d; p-values marked by the letter 'd' indicate performance of the Mann-Whitney test and effect size r; significant at 5%.

## Discussion

This study showed that, overall, for most surfaces monitored using various methods, there was no statistically significant difference between the results from the public and private NICUs. However, regarding visual inspection, it was observed that in the private NICU, four surfaces—the infusion pump (phase I), armchair (phase II), and desk (phases II and III)—showed a statistically significant reduction.

Visual inspection is the most widely used method by professionals, although other methods are available (29, 30). A study conducted in a private general ICU found that, according to the visual inspection method, surfaces such as the infusion pump, alcohol dispenser, and telephone had the highest percentage of accumulated dirt, accounting for 75% of occurrences (31). In contrast, our study in the neonatal ICU found different results, with an approval rate for the infusion pump in phase I of 75% in the private NICU, whereas in the public NICU, the disapproval rate was 75%.

Regarding the ATP method, there was no statistical difference between the two NICUs. However, when analyzing the six surfaces evaluated in the three phases (18 surfaces), it was noted that the absolute median values in relative light units (RLUs) obtained in the public hospital were higher on 12 surfaces, indicating a greater organic matter load. Additionally, the counter surface in the public NICU during phase I had the highest recorded value, with 395 RLUs, while the incubator surface in the private NICU had the lowest median value, at 17 RLUs. A study conducted in an adult ICU found that the telephone surface had the highest median, with 1,012 RLUs, whereas the bed rail had a median of 26 RLUs (31). The surfaces closest to the patient generally had lower median values.

The effectiveness of C&D in NICUs is crucial for preventing HAIs in newborns, particularly in premature and very low birth weight infants. This study compared the microbial count on hospital surfaces and found a significant difference in microbial load only on the scale surface in the private NICU during phase II (*P* = 0.023). In another NICU study, researchers found a median microbial load of 24 CFU, which was significantly reduced to 5 CFU after staff training (32).

A study conducted in an ICU to assess the effectiveness of daily cleaning found that routine cleaning significantly reduced the bacterial load in patient rooms (reduction of 0.14 log_10_ CFU, *P* = 0.008; reduction of 0.21 log_10_ RLU, *P* < 0.001). One key aspect influencing these results was the importance of providing feedback to the team (10). These findings align with the results observed in both public and private NICUs, as phase III showed a reduction in median values compared to phase I.

Regarding the presence of MRSA, there was no statistically significant difference between the public and private institutions. Resistance percentages remained ≤ 50% in phases II and III of the study, except for the scale surface (62.5%) and the armchair (75%) in the public NICU. These results differ from a study conducted in a Moroccan NICU to assess surface C&D, in which 60% of *Staphylococcus aureus* strains were methicillin-resistant (32).

### Continuing education

The literature indicates that interventions often lead to short-term improvements in the C&D process. However, sustaining these improvements over time remains a challenge. One study found that interventions incorporating training, monitoring, and feedback for allied health professionals (AHPs) resulted in improvements lasting more than 10 years (33). In a study conducted in a neonatal care unit, a multimodal intervention—including cleaning audits with feedback, cleaning checklists, in-room cleaning wipes, and training for staff and mothers—led to significant improvements in the C&D process (20).

A study analyzing the monitoring of hospital cleanliness over a decade identified progressive improvements in cleanliness rates. Throughout this period, cleaning rates steadily increased, reaching an average of 93.0% over the last five years. This upward trend reflects the effectiveness of a long-term monitoring program, which enabled continuous adjustments and interventions to maintain and enhance cleaning standards. Additionally, the periodic revision of cleanliness targets, including an updated benchmark of 93%, demonstrates an ongoing commitment to excellence in environmental hygiene. These findings underscore the importance of long-term monitoring as an effective strategy to ensure the consistent quality of hospital cleaning, ultimately yielding tangible benefits for patient safety and wellbeing (33).

### Human factors

The study indicated no statistically significant difference in the self-reported satisfaction and performance of the NPs and HPs responsible for the C&D process. However, it was observed that the median values for satisfaction and performance were higher in the public institution.

The study's findings highlight the participants' job satisfaction. Job satisfaction refers to a worker's perception of their job, which is influenced by personal beliefs, values, interactions with colleagues and the institution, and a sense of belonging (34).

NPs and HPs responsible for C&D in a hospital setting often report higher satisfaction levels regarding supervision and coworkers, while satisfaction is lower regarding salary, benefits, promotion opportunities, and working conditions. In contrast, nurses working in management positions tend to report higher job satisfaction than nursing assistants and technicians (35).

Among professionals involved in the C&D process in hospitals, job dissatisfaction is linked to remuneration, institutional policies, level of autonomy, and professional responsibilities, all of which can contribute to career abandonment. Other factors influencing job satisfaction include gender, age, marital status, education level, living conditions, and external factors such as professional success, career advancement, and the nature of the work performed (36, 37).

Work performance reflects an employee's commitment to implementing the principles and objectives of health programs and policies, which serve as a foundation for maintaining and improving healthcare quality and supporting decision-making in the field (38).

Most participants perceived their work performance and contribution to the institution positively. Training and continuing education initiatives are essential for promoting autonomy and restructuring work processes, which positively impact worker performance. It is recommended that training programs for human resources involved in C&D be developed to mitigate negative health impacts and reinforce environmental preservation. It is also noteworthy that both institutions employ direct-hire staff rather than outsourced workers.

The results observed in NICUs and an Emergency Care Unit (UPA) during the COVID-19 pandemic revealed both similarities and differences regarding human factors and workplace motivation. In the NICU study, no statistically significant differences were found between professionals from the two institutions in terms of age, education level, or length of time working, suggesting a certain homogeneity between the groups. However, in the UPA, a significant association was observed between education level and intrinsic motivation, with individuals with lower educational attainment demonstrating greater motivation linked to work enjoyment (*p* = 0.031). These differences may reflect variations in work environments and the demands of each healthcare setting (29).

Regarding job satisfaction, the NICUs exhibited an interesting distinction: the public institution scored higher on the job performance scale than the private one (*p* = 0.006). In the UPA, satisfaction levels varied significantly, ranging from indifference to agreement with overall job satisfaction. Additionally, female participants in the UPA reported greater satisfaction regarding their work expectations being met (*p* = 0.014), a trend not observed in the NICUs. This comparison suggests that while both environments present challenges, the intrinsic and extrinsic factors influencing motivation and job satisfaction may vary depending on the nature of the job and the specific conditions of each workplace (29).

### Study limitations

Although the study has provided valuable insights into comparing surface C&D in public and private NICUs, some limitations should be considered. The sample was not randomly selected; surfaces were chosen intentionally, which may introduce bias and limit the generalizability of the findings to other NICUs. Additionally, variability in adherence to standardized practices and differences in the use of cleaning products between teams may have influenced ATP and CFU measurements as well as visual inspection results.

## Conclusion

This study emphasizes the importance of implementing and monitoring robust intervention packages for C&D in NICUs, demonstrating that there was no statistically significant difference in most of the monitoring methods used between the two NICUs. The findings suggest that even with differences in institutional settings, the rigorous application of intervention packages resulted in similar outcomes. Furthermore, despite variations in employee performance and job satisfaction levels, the intervention package had a comparable effect on improving C&D practices in both NICUs.

## Data Availability

The raw data supporting the conclusions of this article will be made available by the authors, provided that a reasonable request is made to the corresponding author.
